# *Leishmania braziliensis*-Reactive T Cells Are Down-Regulated in Long-Term Cured Cutaneous Leishmaniasis, but the Renewal Capacity of T Effector Memory Compartments Is Preserved

**DOI:** 10.1371/journal.pone.0081529

**Published:** 2013-11-26

**Authors:** Regina Pereira-Carvalho, Carolina O. Mendes-Aguiar, Manoel P. Oliveira-Neto, Cláudia J. F. Covas, Álvaro L. Bertho, Alda M. Da-Cruz, Adriano Gomes-Silva

**Affiliations:** 1 Laboratório Interdisciplinar de Pesquisas Médicas, Instituto Oswaldo Cruz, FIOCRUZ, Rio de Janeiro, Rio de Janeiro, Brasil; 2 Instituto de Pesquisa Clínica Evandro Chagas (IPEC), FIOCRUZ, Rio de Janeiro, Rio de Janeiro, Brasil; 3 Laboratório de Imunoparasitologia, Instituto Oswaldo Cruz, FIOCRUZ, Rio de Janeiro, Rio de Janeiro, Brasil; Federal University of São Paulo, Brazil

## Abstract

*Leishmania (Viannia) braziliensis* control and tissue damage relate to the effector immune response, which in turn affects clinical outcome. *Leishmania* reactive CD4^+^ and CD8^+^ T cells are expanded in long-term healed cutaneous leishmaniasis (hCL) patients but their functional characteristics remain to be determined. This study investigates antigen-specific recall in long-term healed CL caused by *L. braziliensis* infection. Healed CL subjects were grouped according to the time elapsed since the end of therapy: less than two years and two to five years. Activation phenotype (CD69^+^ or CD25^+^) and subpopulations of memory T cell phenotypes [central memory (Tcm): CD45RO^+^ CCR7^+^ or effector memory (Tem): CD45RO^+^ CCR7^-^] were quantified in *ex vivo* blood mononuclear cells and after *Leishmania* antigens stimuli. A reduction in the percentage of activated *Leishmania*-responder CD4^+^ and CD8^+^ T cells in hCL was associated with the time elapsed since clinical cure. Percentage of CD69^+^ in TCD4^+^ and TCD8^+^ cells were negatively correlated with IL-10 levels. *Ex vivo* analyses showed contracted Tem CD4^+^ and Tem CD8^+^ compartments from hCL with long time elapsed since clinical cure, although renewal of these compartments was observed following *in vitro* exposure to leishmanial stimuli. Our results show that healed *L. braziliensis* infected patients exhibit a recall response to *Leishmania* antigens with evident expansion of effector memory T cells. Regulated leishmanial-specific response seems to emerge only about two years after initial contact with the parasite antigens.

## Introduction

Cutaneous leishmaniasis (CL) due to *Leishmania (Viannia) braziliensis* is characterized by ulcerative lesions in the skin that heal either spontaneously or following therapy. Even if healed patients develop a delayed hypersensitivity reaction to leishmanial antigens, which induce the expansion of *Leishmania*-reactive T lymphocytes, the development of the disease is not always prevented [1-3]. Although lesion healing is accompanied by parasite control and reduction of inflammatory cells in scars [4,5], evidence of parasite persistence [6-9] and leishmanial immune response is still found long after therapy, despite the absence of clinical symptoms [10]. Infections can recidivate not only at the original lesion sites, but also in the mucosal membranes of the upper respiratory tract, resulting in mucosal leishmaniasis (ML). 

Ideally, healed CL patients should be monitored for approximately five years post-therapy to enable early detection of possible relapses or metastatic lesions, as this period is considered critical for disease resurgence [11,12]. Contributing factors to an unfavorable prognosis are, however, still a matter of debate, although evidence suggests that immunological factors are decisive. Indeed, a higher percentage of activated T CD4^+^CD69^+^ and IFN-γ^+^ lymphocytes has been associated with larger lesions, hence more severe disease [13]. Moreover, higher frequencies of CD4^+^ T cells expressing CD28^-^CD69^+^CD62L^low^ are seen in ML than CL cases, along with low regulatory capacity for IFN-γ secretion [14]. These data reinforce the idea of an exacerbated type 1 response in ML subjects, sustained by the replenishment of activated effector cells that may not be down-modulated appropriately, maintaining a persistent inflammatory response and tissue damage. Conversely, an appropriate balance between inflammatory and regulatory factors (ex. IFN-γ/IL-10) might favor parasite control and the stability of clinical cure [15-17]. 

The withdrawing of regulatory T cells (Treg), or associated molecules, results in an exacerbation of the effector immune response [18]. Although previous research has focused mostly on immune responses during the active phase of the disease, investigation following clinical cure has demonstrated a reduction in the activation of effector cells and in the amount of inflammatory mediators [5,10,17,19-23]. These results point to key roles for regulatory mechanisms on sustaining clinical cure. 

Several experimental studies indicate that T-cell memory compartments lead to stronger and faster adaptive immune response against *Leishmania* antigens [24-26]. Indeed, this is the basis for the design of vaccines against infectious organisms [27]. However, whether anti-*Leishmania* memory remains after parasitological cure still needs to be determined [24-26,28-30]. Recently, *Leishmania*-reactive proliferating effector memory TCD8^+^ were identified as the most frequent subset cells in cured *L. major* or *L. tropica* leishmaniasis patients from Iran, which suggested their role in recall immune response [31]. However, the characterization of Tcm (CD45RO^+^CCR7^+^) and Tem (CD45RO^+^CCR7^-^) induced by L. (V.) *braziliensis* infection and the magnitude of T cell recall long term after cure are not known. These features are of crucial importance for understanding the ultimate protective immune response.

The present study investigates antigen-specific recall in long-term healed CL caused by infection with *L. braziliensis*. Such capacity was examined by *ex vivo* leishmanial-antigen stimulation to determine the renewal of memory T cell compartments and replenishment of phenotypically defined CD4^+^ and CD8^+^ T cells. Our previous results suggest that qualitative and quantitative changes in immune response can occur in leishmaniasis patients over time: a higher proportion of leishmanial-reactive CD4^+^ than CD8^+^ T cells were observed in long-term cured patients and, although this profile was similar to active disease [10], it was characterized by lower cell percentages. Our hypothesis is that long-term effector memory T cell compartments enable the activation of cellular immunologic pathways under *L. braziliensis* stimulus. Because determining the T cell compartment that is preferentially expanded to control the *Leishmania* infection is critical to understand protective immune responses, our results represent a step forward towards the design of an effective vaccine to control this disease, currently considered one of the priority endemic diseases of the world [32]. 

## Materials and Methods

### Ethics statement

This study was approved by the ethics committee of the Instituto de Pesquisa Clinica Evandro Chagas (IPEC), Ministério da Saúde, Brazil. It abides by the Helsinki Declaration on human subject research. Written consent was obtained from all volunteers and the study protocol was approved by the Ethical Committee of IPEC (n° 069/2008).

### Study population

Twenty-six subjects (12 males, 39.7±16.9 years old) with healed cutaneous leishmaniasis (hCL), as defined by past CL diagnosis and absence of recurrent lesions, were enrolled in the study. Patients were from areas in Rio de Janeiro (Brazil) where *L. braziliensis* is endemic. Clinically healed lesions were defined as scars presenting a complete re-epithelialization with absence of hyperemia, oedema, and desquamation. Subjects were treated according to the guidelines of the Brazilian Ministry of Health (15-20 mg/kg/day of Sb^+5^ for 20-30 days) and were followed-up for five years at IPEC/FIOCRUZ. Enrolled individuals were sub-divided into two groups, depending on the time elapsed since the end of therapy: less than two years (hCL< 2y, n=13, 40.6±18.5 years old, 8 males) or from two to five years (hCL 2-5 y, n=13, 38.6±14.4 years old, 10 males). Healthy subjects (HS, n=12, 26 ± 5.7 years old, 5 males) had no clinical or epidemiological evidence of *Leishmania* infection, as well as negative lymphocyte proliferative responses to *L. braziliensis* antigens. 

### *In vitro* stimulation of peripheral blood mononuclear cells with *Leishmania braziliensis* antigens

Peripheral blood mononuclear cells (PBMC) were separated by centrifugation over a gradient of Ficoll-Hypaque (Histopaque 1077; Sigma Chemical Company, St Louis, MO, USA). PBMC (3 x 10^6^ cells per mL) were cultured *in vitro* in 24-well flat-bottom plates (Nunc, Roskilde, Denmark) in the presence of 5 x 10^6^ (50 µg/well) disrupted *L. braziliensis* (MHOM/BR75/M2903) promastigotes (Lb-Ag). Cells cultured for five days at 37°C in a humidified atmosphere of 5% CO_2_ in air were harvested and lymphocyte proliferation response (LPR) measured by ^3^H-thymidine incorporation, as described previously [10]. Culture supernatants were collected and stored at −20°C until cytokine levels were measured. 

To conduct the phenotypic analysis, *ex vivo* PBMC and *in vitro* culture cells were stained with fluorescein isothiocyanate-labelled anti-CD4, phycoerythrin-Cy5-labelled anti-CD8 (Immunotech, Beckman Coulter Corporation, Marseille, France), phycoerythrin-labelled anti-CD25 or anti-CCR7, and phycoerythrin-Cy7 labelled anti-CD69 or anti-CD45RO (Becton Dickinson Bioscience Pharmingen, Franklin Lakes, NJ, USA). Forty thousand events were acquired from each sample into a lymphocyte gate using a Cyan flow cytometer (Beckman Coulter Inc., FL, USA). Surface molecules were analyzed using the Summit 4.3 software (DakoCytomation, Fort Collins, CO, USA). Lymphocyte populations were defined by gating on CD3^+^ cells. The frequency of positive cells was analyzed in two regions: lymphocyte gate (*ex vivo* PBMC) and large lymphocyte blast gate (*in vitro* antigen stimulated PBMC). The limits for the quadrant markers were always set based on negative populations and isotype controls. Results are expressed as percentage of positive cells.

### IL-10 cytokine measurement

Levels of IL-10 were measured in culture supernatants by ELISA according to the manufacturer’s instructions (R&D Systems, Emeryville, CA, USA). Samples were tested in duplicate and their concentration analyzed using the SOFTmax®PRO 4.0 program (Life Sciences Edition, Molecular Devices Corporation, USA). The sensitivity of the assay ranged from 31.25 to 2,000 pg/mL.

### Statistical analysis

Because the data were not normally distributed, Kruskal-Wallis with post-test Dunns, to compare selected pars of columns, and Spearman rank correlations were performed using GraphPad Prism version 5.0 for Windows (GraphPad Software, San Diego, CA, USA). Notice that sample size differences were due to differences in the number of cells obtained for the experiments.

## Results

### Healing duration is inversely related to T cell activation levels

**Figure 1:**
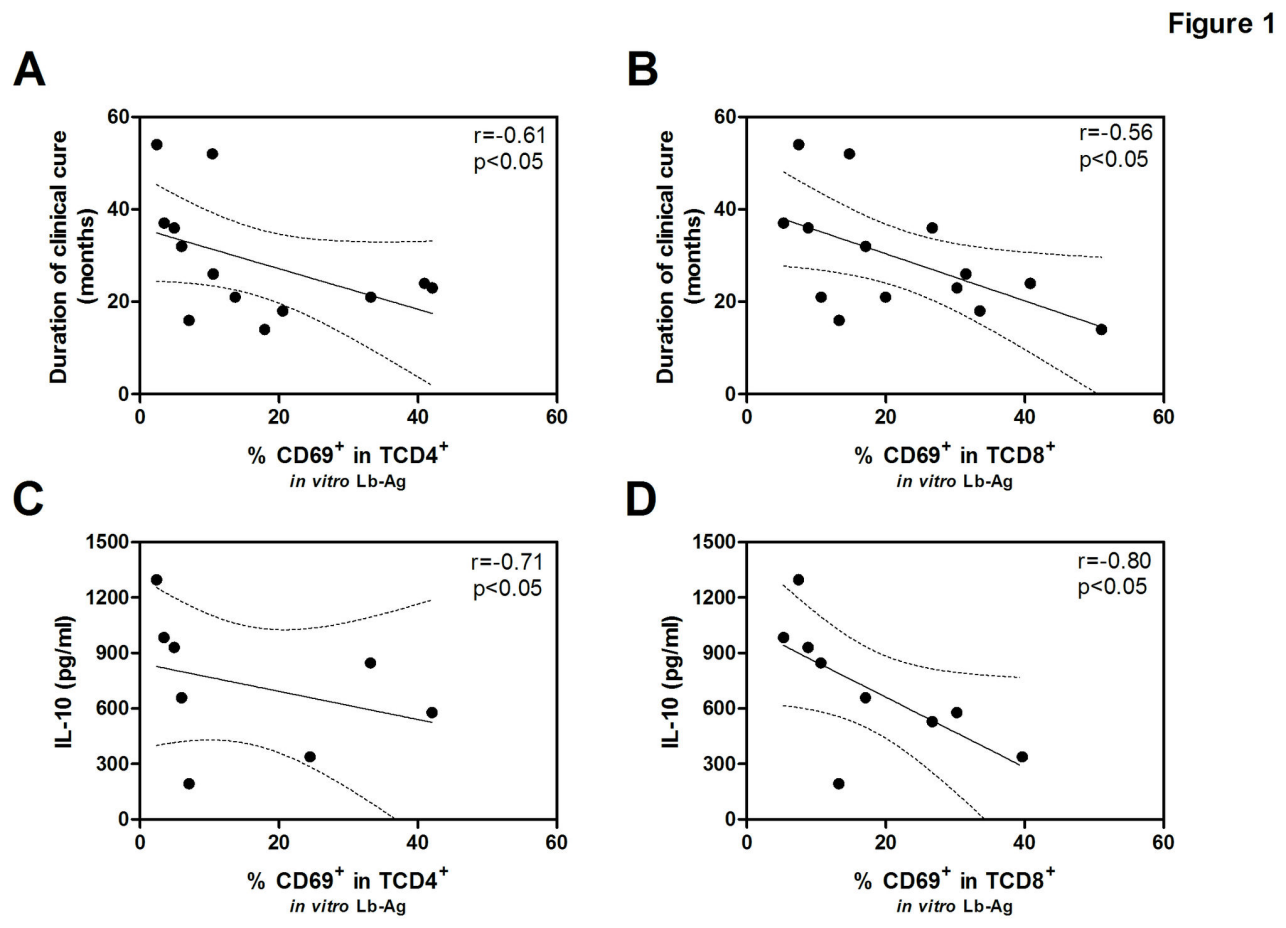
Correlation analysis of clinical and/or immunological parameters from healed cutaneous leishmaniasis subjects. Correlation between the percentage of recently activated CD4^+^ (A, n=13) or CD8^+^ (B, n=14) T lymphocytes from PBMC after *in* *vitro* Lb-Ag stimulation and the duration of clinical cure; Correlation between the percentage of activated CD4^+^ (C, n=8) or CD8^+^ (D, n=9) T lymphocytes and the concentration of IL-10 from cell culture supernatant; Each point represents one subject. The graphs show the best fitted lines with 95% confidence intervals. r= correlation coefficient; p= significance level, (Spearman test).

The percentage of CD69^+^ and CD25^+^ *ex vivo* and in *Leishmania*-reactive T CD4^+^ and TCD8^+^ cells in the two groups (hCL<2y and hCL>2-5y) is shown in Table 1. The *ex vivo* percentage of TCD4^+^ and TCD8^+^ cells presenting CD69 on the cell surface membrane was higher in recently cured patients than in healthy subjects (HS) (p<0.01 and p>0.05, respectively). In the hCL<2y group, Lb-Ag stimuli of PBMC increased the CD69^+^ percentage in TCD4^+^ (8.9-fold increase) and TCD8^+^ (8.1-fold increase) cells when compared to non-stimulated cells. The same was observed for, CD25^+^CD4^+^ (3.6-fold increase) and CD25^+^CD8^+^ (10-fold increase). However, CD69 levels in those patients in the hCL 2-5y group were not significantly different from those of HS, except for CD69^+^ in TCD8^+^. Accordingly, activation levels in Lb-Ag stimulated cells were inversely associated with duration of clinical cure, as observed for CD69^+^ in TCD4^+^ (*r*= -0.61, p<0.05, n=13), CD69^+^ in TCD8^+^ (*r*= -0.56, p<0.05, n=14) (Figures 1A and 1B, respectively), and CD25^+^ in TCD8^+^ (*r*= -0.69, p<0.001, n=22), but not for CD25^+^ in T CD4^+^ (*r*= -0.28, p>0.05, n=21).

**Table 1:** Comparative analysis of the percentage of activated T lymphocytes prior to and following *Leishmania* antigen stimulation in healed cutaneous leishmaniasis groups (hCL<2years: period since end of therapy lower than 2 years; hCL 2-5years: period since end of therapy from two to five years) and healthy subjects.

	**hCL < 2 years**				**hCL 2-5 years**				**Health Subjects**		
T	*Ex vivo*	*In vitro*	Fold	P – value **^*c*^**	*Ex vivo*	*In vitro*	Fold	P – value **^*c*^**	*Ex vivo*	*In vitro*	Fold
lymphocyts	PBMC	Lb-Ag	increase **^*b*^**		PBMC	Lb-Ag	increase **^*b*^**		PBMC	Lb-Ag	increase **^*b*^**
		stimulated				stimulated				stimulated	
**%CD69^*+*^**	6.3**^*a*^**	20.5	8.9±2.9	<0.05	1.3	5.4	2.7±2.4	n.s.	1.1	1.4	1.5±1.1
**in T CD4^+^**	3.7 - 30.7	13.7 - 40.9			0.8 - 2.5	3.2 - 10.4			0.6 - 2.9	1.1 - 5.6	
	n=7	n=7			n=8	n=6			n=8	n=7	
**%CD69^*+*^**	5.9	30.2	8.1±6.3	<0.01	1.9	14.7	2.6±2.3	<0.05	2.5	2.9	1.5±1.4
**in T CD8^+^**	2.0 - 8.3	13.3 - 40.8			0.9 - 3.0	7.5 - 26.7			1.7 - 3.6	2.1 - 5.1	
	n=7	n=7			n=8	n=7			n=8	n=7	
**%CD25^*+*^**	9.4	40.9	3.6±1.7	<0.05	8.1	17.2	3.0±1.7	n.s.	9.5	10.2	1.4±0.7
**in T CD4^+^**	8.0 - 15.3	19.4 - 50.4			6.3 - 9.5	10.6 - 43.4			6.1 - 13.7	7.3 - 12.3	
	n=10	n=11			n=13	n=10			n=12	n=10	
**%CD25^*+*^**	2.9	19.5	10.0±6.9	<0.01	1.4	5.5	1.9±1.7	n.s.	1.2	1.6	2.6±1.8
**in T CD8^+^**	1.2 - 10.0	10.5 - 55.4			0.7 - 2.5	3.7 - 8.7			0.8 - 2.3	1.2 -8.5	
	n=10	n=11			n=13	n=11			n=12	n=10	

*a* Data expressed as median, 25th and 75th percentile and number of subjects evaluated.

*b* Fold increase between *in vitro* Ag-Lb stimulated and non-stimulated cells. Values indicate the mean ± standard deviation.

*c* Statistical significance of the difference in the percentage of activation of *in* *vitro* *Leishmania braziliensis* *antigens* (Lb-Ag) stimulated cells between the hCL groups (hCL<2years or hCL 2-5years) and healthy controls (HS), as determined by Kruskal-Wallis with post-test Dunns to compare selected pars of columns.

n.s. - not significant.

### Leishmania-activated T cell levels may be down-modulated by regulatory functions

As the percentage of CD69^+^ T cells induced by leishmanial antigens is lower in long-term healed CL patients, we examined whether regulatory factors could be associated with reduced lymphocyte activation. The results show a negative correlation between IL-10 levels following Lb-Ag stimuli *in vitro* and the percentage of T cells activated in terms of CD69 expression both in TCD4^+^ (*r*= -0.71, p<0.05, n=08) and TCD8^+^ cells (*r*= -0.80, p<0.05, n=09) (Figures 1C and 1D, respectively), indicating that IL-10 may play a role in controlling cell activation status. 

### The ability to renewal effector memory T cells is maintained long after cutaneous leishmaniasis healing

**Figure 2:**
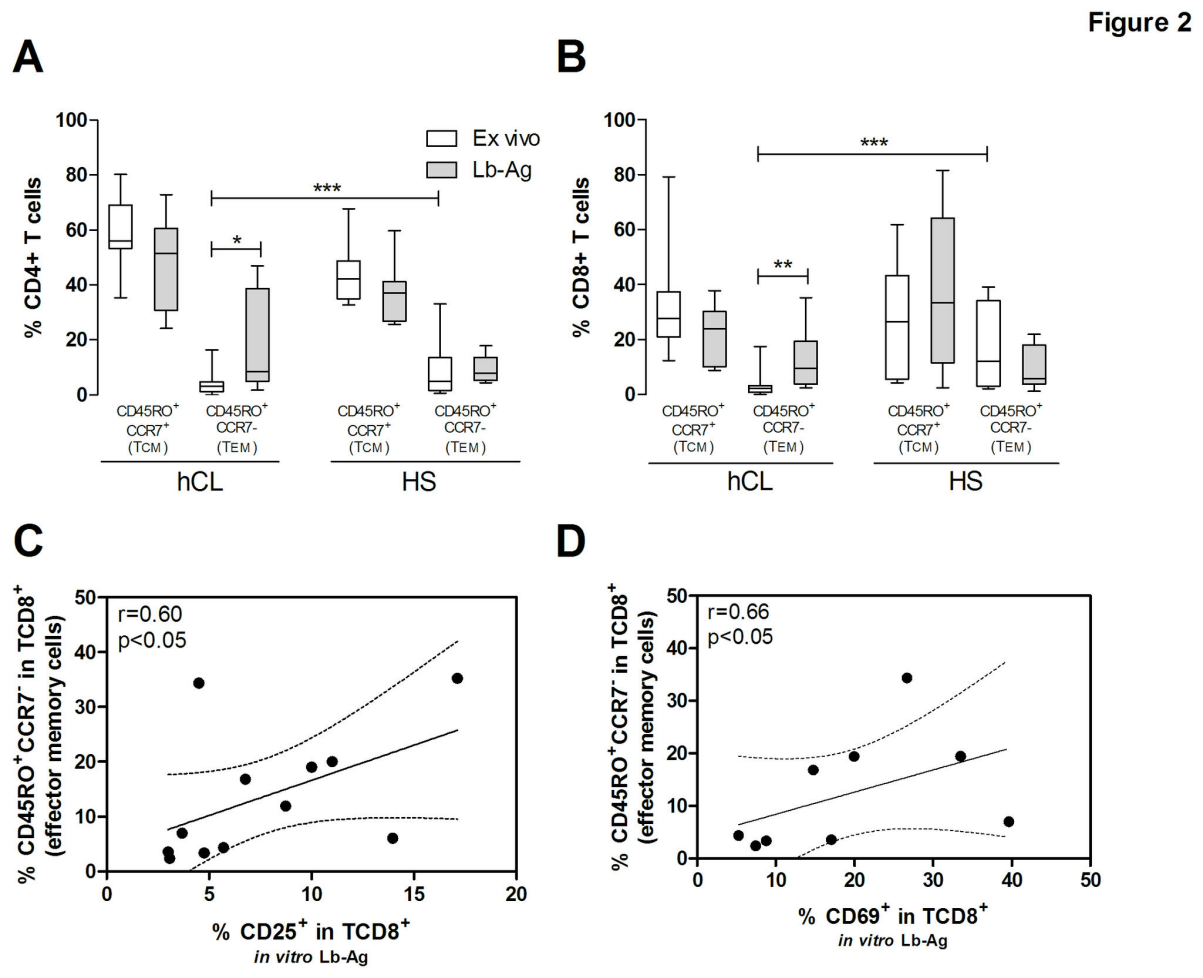
Analysis of renewal capacity of memory compartments and activated T cell replenishment of healed cutaneous leishmaniasis subjects. Renewal capacity of CD4^+^ (**A**) and CD8^+^ (**B**) T cells phenotipically characterized by central memory (Tcm, CD45RO^+^CCR7^+^) or effector memory (Tem, CD45RO^+^CCR7^-^). The white box and whiskers represent those cells evaluated immediately after PBMC (*ex vivo*) and the gray boxes represent those cells after *in* *vitro* stimuli with Lb-Ag. The central line represents median values. hCL= healed cutaneous leishmaniasis; HS= healthy subjects; *p<0.05, **p<0.01, ***p<0.001 (Kruskal-Wallis with post-test Dunns). Correlation analysis between the percentage of activated CD25^+^ (**C**, n=9) or CD69^+^ (**D**, n=12) in CD8^+^ T cells after *in* *vitro* Lb-Ag stimuli and the percentage of effector memory (CD45RO^+^CCR7^-^) CD8^+^ T cells after *in* *vitro* Lb-Ag stimuli. Each point represents one subject. The graphs show the best fitted lines with 95% confidence intervals. r= correlation coefficient; p= significance level, (Spearman test).

To determine if the progressive reduction of *Leishmania*-activated T cells was due to a putative loss of T cells able to recognize these antigens, central (Tcm) and effector (Tem) memory compartments were investigated. Tcm (CD45RO^+^CCR7^+^) and Tem (CD45RO^+^CCR7^-^) frequency from hCL with long time elapsed since clinical cure were analyzed *ex vivo* and after *in vitro* Lb-Ag stimuli. *Ex vivo* analyses show that these patients exhibit much lower percentages of Tem CD4^+^ (4.4%±4.4%, median=3.1%, n=15, p<0.001) and Tem CD8^+^ (3.3%±4.2%, median=2.3%, n=15, p<0.001) than HS (Figures 2A and 2B, respectively). However, a significant increase in the percentage of Tem CD4^+^ (18.6%±17.6%, median=8.4%, n=12, p<0.05; Figure 2A) and Tem CD8^+^ cells (13.7%±11.7%, median=9.4%, n=12, p<0.01; Figure 2B) was observed following exposure to leishmanial antigen. The results also show that renewal capacity was highly heterogeneous for hCL subjects, with two patients presenting much higher levels of Tem CD8^+^ (34.4% and 35.2%) than the median (9.4%). 

The percentage of Tem CD8^+^ renewed in response to leishmanial stimuli was positively associated with the level of antigen-activated CD8^+^ T cells (%CD25^+^ in TCD8^+^: r= 0.60, p<0.05, Figure 2C and %CD69^+^ in TCD8^+^: r= 0.66, p<0.05, Figure 2D), indicating that Tem CD8^+^ cells contribute to the generation of activated CD8^+^ T cells, with a possible effector function.

## Discussion

T cell recall response to *Leishmania* antigens developed by long-term healed CL patients can mimic, at least partially, a possible re-exposure to this parasite due to re-infection or persistent parasite replication. In both cases, activation of specific memory clones is induced. Although previous studies have shown that *Leishmania* reactive CD4^+^ and CD8^+^ T cells are expanded in long-term cured patients, the functional characteristics of these cells remain to be determined. The present results indicate a reduction in the percentage of activated *Leishmania*-responder CD4^+^ and CD8^+^ T cells in healed CL patients, which depends on the time elapsed since clinical cure. Additionally, we have shown that such reduction might be associated with an increased participation of regulatory mechanisms in this process. Surprisingly, *ex vivo* analyses of peripheral blood show contracted Tem CD4^+^ and Tem CD8^+^ compartments in hCL patients, although renewal of these compartments was also observed following *in vitro* exposure to *Leishmania* stimuli. Finally, this increase of Tem CD8^+^ under Lb-Ag stimuli was positively correlated with the frequency of activated TCD8^+^ cells, but was not associated with cytotoxity phenotype.

Previous research has shown that *L. braziliensis* infection affects the activation of circulating T lymphocyte subtypes [13,14,33]. Here we show that healed CL patients maintain high levels of *ex vivo* activated TCD4^+^ and TCD8^+^ cells compared to HS controls. T cell activation levels only return to baseline (control) values approximately two years after the end of therapy. Indeed, except for the percentage of CD25^+^ in CD4^+^ T cells, CD4^+^ and CD8^+^ T cell activation levels are negatively associated with the duration of clinical cure. These data show that although disease is apparently limited to the skin, cells from other compartments (such as blood and lymphoid organs) are also affected, as similarly observed in non-human primates [23]. Additionally, the low frequency of *Leishmania* responder lymphocytes in the blood compared to that in lesions [34,35] suggests that both specific and nonspecific cells may be systemically activated, possibly by soluble factors released from *Leishmania* stimulated cells [36,37]. Our findings show that these phenotypic alterations take longer to return to normal status. Similarly, discrete to moderate inflammatory infiltrates are still detected in 1- and 3-year-old CL scars, reinforcing the idea that complete resolution of pathological damage also takes longer to occur [5]. 

An alternative possibility is that reminiscent parasites in the lymphonodes act as a source of antigenic stimuli, as observed in HIV infection [38]. We examined this possibility by investigating how circulating T cells responded to *Leishmania* antigens. Although a significant increase in CD69^+^ T cell subsets was observed for long-term hCL patients compared to healthy individuals, the increase of antigen-activated T cells was lower in the latter group than in recently cured individuals. This reduced T cell activation was to be expected, as high activation levels are associated with greater disease severity, including the occurrence of larger cutaneous lesions [13] and mucosal disease [14]. The reduction in parasite burden after therapy and consequent decrease of *Leishmania*-antigen cell stimulation has been indeed suggested to contribute to a homeostatic immune ambiance.

The magnitude of effector T cell responses can be controlled by regulatory T cells at the lesion site by suppressing lymphocyte proliferation [39]. For example, antigen-induced FoxP3 and IL-10 have been associated with an increased risk of cutaneous lesions in populations exposed to *L. braziliensis* [40]. Conversely, low levels of IL-10 and absence of IL-10 receptor are related to exacerbated immune responses in mucosal leishmaniasis [16,41,42], suggesting that a failure in the regulatory mechanism that down-modulates effector responses may lead to mucosal damage. Our results show higher IL-10 levels in cured patients presenting lower activation levels. Accordingly, the recruitment of Treg cells (CD4^+^CD25^+^IL-10^+^) has been reported in healing tissues of non-human primate models infected with *L. braziliensis* [23]. 

Memory T cell responses guarantee a rapid secondary reaction in re-exposure to *Leishmania* infection either by reinfection or persistent *Leishmania* replication [27,29]. This is the basis of immune protection against infectious diseases. In this study, although blood Tem compartment size in HS was consistent with that previously reported [43,44], blood Tem from long-term hCL was contracted, even more than two years following therapy. This is an unexpected result, especially considering the normal tendency of Tem expansion in successively experienced immune response [31,45]. As this phenomenon was seen in blood lymphocytes, it indicates that those cells that are specific for all other antigens, not *Leishmania*-specific, are globally affected. In contrast, patients cleared from filarial infection presented blood Tem levels similar to those of healthy subjects [44]. In addition, there were no age differences between early and late hCL individuals (a factor that could potentially affect these memory compartments) [45]. Further research is therefore needed to examine putative mechanisms that would enable *Leishmania* infection to affect the size of the effector memory compartment even long after the infection has been controlled. 

Lymphocytes from long-term healed CL recognize leishmanial stimuli and proliferate upon exposure. Although blood Tem CD4^+^ and Tem CD8^+^ compartments were contracted, our results show that they can be renewed after a secondary challenge with parasite antigens, as suggested by the association between the increase in Tem and the levels of activated TCD8^+^. Moreover, leishmanial induced IFN-γ production is maintained in long-term healed CL [10,16]. However, it is interesting to take account that in murine model memory T cells can be maintained even in the absence of persistent *L. major* infection [24]. Altogether, these results indicate a capacity to generate anti-*Leishmania* immune effector mechanisms. Maintenance of this protective immune response along with regulatory effects is of paramount importance, since long-term hCL individuals cannot achieve sterile cure, once parasite persistence was detected in the blood [7,8], scars [6] and in lymph nodes of clinically cured subjects [46]. Two patients have also presented high levels of induction of antigen reactive Tem CD8^+^, pointing to a possible generation of exacerbated effector responses predisposing to mucosal disease [47]. While constant parasite stimulus leads to benefic induction of immunological memory, it can also contribute to chronic maintenance of activated effector cells. In addition to a poor regulatory response, exacerbated TCD8^+^ activity could, however, underlie an unfavorable fate with regard to ML [16,17,41,42,47,48]. 

One limitation of this study derives from the analysis of cells responsive to several *Leishmania* antigens, as opposed to the analysis of lymphocytes specific to dominant epitopes in viral infections [49,50]. Regardless, our results clearly showed that after stimuli with leishmanial antigens it was observed a frequency augment of activated lymphocytes or effector memory T cells derived from hCL. However, this study provides evidence of the presence of memory compartments in human leishmaniasis resulting from infection by *L. braziliensis*. 

In conclusion, our results show that healed *L. braziliensis* infected patients exhibit a recall response to *Leishmania* antigens with evident expansion of effector memory T cells. They also indicate that regulatory mechanisms favor a down-modulation of activation status, which can prevent exacerbated immune responses without the loss of protective immunity. Which memory cell compartment should be preferentially expanded by a vaccine candidate is a crucial matter for discussion. The fact that we still do not have a vaccine effective against parasitic infections that rely on cellular immune response, and the finding that regulated leishmanial-specific response will emerge only about two years after initial contact with the parasite antigens, lead to the question: are clinical trials aimed at determining vaccine efficacy being conducted too early? 
